# Steindler flexorplasty to restore elbow flexion in C5-C6-C7 brachial plexus palsy type

**DOI:** 10.1186/1749-7221-2-15

**Published:** 2007-07-11

**Authors:** Ricardo Monreal

**Affiliations:** 1 "Manuel Fajardo" Teaching Hospital. Orthopedics and Traumatology Department. Zapata y calle D, Vedado, CP: 10400, Havana, Cuba

## Abstract

**Background:**

Loss of elbow flexion due to traumatic palsy of the brachial plexus represents a major functional handicap.

Then, the first goal in the treatment of the flail arm is to restore the elbow flexion by primary direct nerve surgery or secondary reconstructive surgery.

There are various methods to restore elbow flexion which are well documented in the medical literature but the most known and used is Steindler flexorplasty.

This review is intended to detail the author's experience with Steindler flexorplasty to restore elbow flexion in patients with brachial plexus palsy C5-C6-C7 where wrist extensors are paralyzed or weakened.

**Methods:**

We conducted a retrospective follow-up study of 12 patients with absent or extremely weak elbow flexion (motor grade 2 or less), wrist/finger extensor and triceps palsy associated; who had undergone surgical reconstruction of the flail upper limb by tendon transfer (Steindler flexorplasty) and wrist arthrodesis to restore elbow flexion. The aetiology of elbow weakness was in all patients brachial plexus palsy (C5-C6-C7 deficit). Data were collected from medical records and from the information obtained during follow-up visits.

Age, sex, preoperative strength (rated on a 0 to 5 scale for the flexors of the elbow, wrist flexors, pronator and triceps), previous surgery, length of follow-up, other associated operative procedures, results and complications were recorded.

**Results:**

The results are the follows: Eleven patients were found to have very good or good function of the transferred muscles. One patient had mild active flexion of the elbow despite the reconstructive procedure.

There were no major intraoperative complications. Two patients experienced transient, intermittent nocturnal ulnar paresthesias postoperatively. In both patients these symptoms subsided without further surgery.

**Conclusion:**

Our study suggests that in patients with C5-C6-C7 palsy where the wrist and finger extensors are paralyzed or weaked, the flexor-pronators muscles of the forearm are strong but the triceps is not available for transfer; Steindler flexorplasty to restore elbow flexion should be complemented with wrist arthrodesis.

## Background

Traction injury of the brachial plexus results in partial or total paralysis of the upper limb, especially when there is paralysis of elbow flexion. Good hand function is wasted if the hand cannot be maintained in a useful position.

Loss of elbow flexion due to traumatic palsy of the brachial plexus represents a major functional handicap.

Then, the first goal in the treatment of the flail arm is to restore elbow flexion by primary direct nerve surgery or secondary reconstructive surgery.

There are various methods to restore elbow flexion which are well documented in the medical literature. One of the earliest procedures for restoring function to the elbow, Steindler flexorplasty first reported in 1918 [1], is still preferred by many surgeons.

This review is intended to detail the author's experience with Steindler flexorplasty to restore elbow flexion in patients with brachial plexus palsy C5-C6-C7 where wrist extensors, fingers extensors and triceps are paralyzed or weakened.

## Methods

We conducted a retrospective follow-up study of 12 patients with absent or extremely weak elbow flexion (motor grade 2 or less), wrist/finger extensor and triceps palsy associated; who had undergone surgical reconstruction of the flail upper limb by tendon transfer (Steindler flexorplasty) and wrist arthrodesis to restore elbow flexion. The aetiology of elbow weakness was in all patients brachial plexus palsy (C5-C6-C7 deficit). Data were collected from medical records and from the information obtained during follow-up visits.

Five of the patients in this series had been previously treated by surgical exploration with neurolysis, nerve grafting or nerve transposition. At the time of tendon or muscle transfer no patient was considered a candidate for additional nerve exploration or grafting.

**Figure 1:**
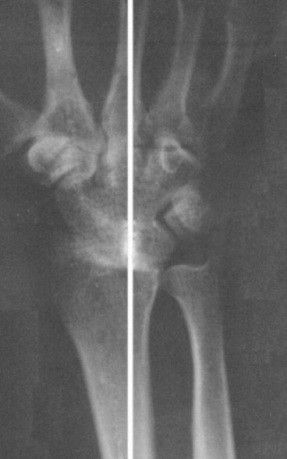
Wrist arthrodesis performed with the single-intramedullary-rod technique.

The wrist was fused in a position that will not be fatiguing and that will allow maximum grasping strength in the hand. This is usually one of 10° to 20° extension, the long axis of the second or third metacarpal shaft being aligned with the long axis of the radial shaft (Figure 1).

**Figure 2:**
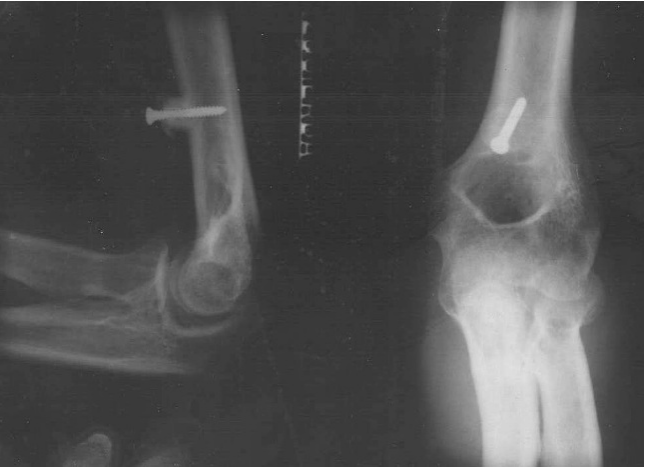
Steindler flexorplasty: proximal transfer (4 – 5 cm) and fixation of a piece of the medial epicondyle with its attached origin of the flexor-pronator muscle group in the middle of the anterior aspect of the humerus.

When a solid wrist fusion is obtained (usually about 12 weeks) a tendon transfer (Steindler flexorplasty) is mandatory. Two points must be emphasized with regard to this procedure: (1) Powerful activity of the flexor-pronator forearm muscles and (2) proximal transfer (4–5 cm) and fixation of a piece of the medial epicondyle (less than one centimetre in thickness) with its attached origin of the flexor-pronator muscle group in the middle of the anterior aspect of the humerus. (Figure 2)

Age, sex, preoperative strength (rated on a 0 to 5 scale for the flexors of the elbow, wrist flexors, pronator, flexor digitorum superficiales and triceps), previous surgery, length of follow-up, other associated operative procedures, results and complications were recorded (Table 1).

**Table 1:** Data on the flexorplasties evaluated.

Case	Age and Sex	Pre operative Strength*					Length of Follow-up (*months*)	Previous Surgery	Associated Procedures	Results	Complications
		*Elbow flexors*	*Wrist flexors*	*FDS*	*Pro*	*Triceps*					

1	34/M	2	5	5	5	1	54		TT	VG	
2	43/F	1	4	4	3	2	18	nl, ng, nt		G	
3	19/M	0	5	5	4	3	7		SA	G	
4	21/M	1	5	5	5	2	51	ng, nt		VG	
5	35/M	1	4	4	5	0	9			VG	UP
6	18/M	0	5	5	4	1	4		TT	G	
7	21/M	2	5	5	5	0	13	nl, ng	SA	VG	
8	20/M	0	4	5	4	0	46		TT	M	
9	52/M	1	5	5	5	2	62			VG	UP
10	21/M	1	4	4	5	2	31	ng, nt	TT	VG	
11	26/F	2	5	5	4	3	14			VG	
12	16/M	2	5	5	5	1	24	nl		VG	

* Strength rated on a scale of 0 to 5

nl: neurolysis; ng: nerve grafting; nt: neurotisation

VG: very good; G: good; M: mild; F: fair

TT: Trapezius Transfer

SA: ShoulderArthrodesis

UP: Ulnar Paresthesias

FDS: Flexor digitorum superficiales

Pro: Pronators

Flexion was measured with a goniometer from the position of complete extension so that 0° of flexion equalled completed extension. In the examination of patients with shoulder fusion or trapezius transfer, care was taken to prevent the patient from using the shoulder to change position of the elbow.

Functional improvement was scored using the criteria established by Alnot and Abols [2]. Two aspects must be kept in mind to evaluate the results obtained after Steindler flexorplasty: muscular power and range of motion.

Very good: Active elbow flexion against resistance (Grade 4) and range of flexion 120°.

Good: Active elbow flexion against resistance (Grade 4) and range of flexion below 120°.

Mild: Active elbow flexion against gravity but not resistance (Grade 3) and range of flexion 80° or more.

Fail: No active elbow flexion against gravity (Grade 0 to 2).

Additional operative procedures were performed to enhance function of the extremity. These included two shoulder arthrodesis and four trapezius transfers to treat flail shoulder. (Table 1)

## Results

The average duration of clinical follow-up was 27.8 months, with a range from 4 months to 62 months. There were two female and 10 male patients with an average age at operation of 27.2 years (range 21 to 52 years).

Preoperatively, all patients had powerful activity of the flexor-pronator forearm muscles, two patients had active elbow extension grade 3 and 10 patients had no detectable active extension of the elbow (grade 2 or less).

**Figure 3:**
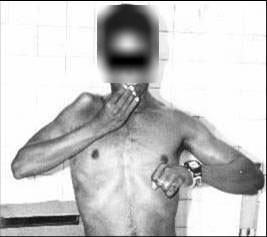
Case 7, after flexorplasty of the right elbow and arthrodesis on the right shoulder. Active elbow flexion to 135 degrees (Very good result) allows positioning of the hand to the head and face for independent self-care.

The patients' assessments of the outcome showed that 11 patients were found to have very good (Figure 3) or good function of the transferred muscles and one patient had mild active flexion of the elbow despite the reconstructive procedure. (Table 1)

There were no major intraoperative complications. Two patients experienced transient, intermittent nocturnal ulnar paresthesias postoperatively. In both patients these symptoms subsided without further surgery. There were no wound infections or and no losses of fixation of the transfer.

## Discussion

Many patients with brachial plexus injuries can be benefited by neurolysis nerve grafting and neurotization procedures. Loss of elbow flexion after traumatic brachial palsy represents a major functional handicap. Although a direct approach to the neurological lesion has given some encouraging results, these can be incomplete and for this reason tendon transfers still have an important role. The use of muscle transfer depends on the individual patient but should always be part of an integrated program that includes nerve repair and muscle transfers.

Restoration of active elbow flexion is the chief concern in the patient with upper or total brachial plexus paralysis. Restoration of active elbow flexion should be considered in most cases as a higher priority than shoulder function.

There are various methods to restore elbow flexion which are well documented in the medical literature. These reconstructive procedures include proximal transfer of the forearm flexor-pronator or wrist extensor mass [1-4] anterior transfer of the triceps tendon [5-7] pectoralis major transfer [8-10] latissimus dorsi transfer [11-13] transfer of the flexor carpiulnaris [14] transfer of the sternocleidomastoid with or without shoulder arthrodesis [15] and free muscle transfer [16].

Anterior transfer of the triceps tendon was designed for patients in whom paralysis or injury had left the flexor-pronator mass unusable for transfer.

Transfer of the pectoralis major muscle to the biceps tendon requires a grade of at least 4 of 5 for the strength of the sternal head of the pectoralis major muscle.

Transfer of the latissimus dorsi muscle also requires a grade of 4 of 5 for the strength of the donor muscle and is technically demanding but results in moderate elbow flexion superior to that seen with other techniques.

A free microneurovascular muscle transfer may be performed in a limited number of patients. The most commonly selected donor muscle is the gracilis. This procedure is indicated when no functional muscles are available for transfer.

The type of muscle transfer depends on the muscle groups remaining available. Alnot [17] recommended triceps transfer in cases of triceps-biceps co-contractions, and Steindler's procedure when the elbow flexors reach only grade 2, contrarily it is contraindicated when the elbow flexors are classified as grade 0, when the wrist flexors are weak, or when wrist and finger extensors are paralyzed. Sometimes it is recommended to complete Steindler flexorplasty by a pectoralis minor transfer in some C5-C6-C7 palsies.

In general proximal advancement of the forearm flexor/pronator muscle group should be considered as the initial treatment in all patients because of its simplicity and lack of donor deficit. According to Segal, Seddon, and Brooks [18], when the pattern of paralysis is such that a free choice of procedures is possible, the Steindler flexorplasty is preferable. Carroll [6] advises against transferring a muscle arising from the medial epicondyle to restore hand function until after any indicated flexorplasty has been done and the strength and function of the transferred muscles have been regained. A modified Steindler flexorplasty was used by Chen W. [19] to restore elbow flexion in 8 patients with post-traumatic flail elbow and the results were not compromised in patients whose flexor tendons had been transferred for wrist and finger extension.

Brunelli GA. et al [20] recommend a modified Steindler procedure to restore elbow flexion. The modifications were designed to avoid the phenomenon of the patient having to make a fist in order to obtain elbow flexion (Steindler's effect). According with the authors, Steindler flexorplasty is indicated in upper plexus lesions (C5-C6); other transfers are more appropriate for lower plexus palsies.

There is general agreement regarding the efficacy of the Steindler flexorplasty. Despite varying criteria, a number of authors [1-4] have reported a 70 to 90 per cent success rate with this procedure. We agree that it is the procedure of choice for a patient who has paralysis of the biceps brachii and brachialis, a functional hand, and sufficient forearm flexor power to warrant transfer.

Proximal transfer of the medial epicondylar muscles is an important adjunct in the rehabilitation of the paralyzed upper extremity, when there is adequate power of this muscle mass and full extension of the elbow is not required for transfer or ambulation. When the medial epicondylar muscles are weak or full extension of the elbow is essential for transfer or ambulation, an alternative procedure must be considered.

An isolated lesion of C7 does not cause a complete muscular paralysis because the proximal muscles innervated by C7 are also innervated by C6 (pronator teres, teres major, flexor carpi radialis, triceps, extensor indicis and digiti minimi proprii) while the lower muscles innervated by C7 are also innervated by C8 (palmaris longus, triceps, extensor carpi ulnaris, abductor pollicis longus, extensor pollicis brevis, extensor pollicis longus, flexor carpi ulnaris). The sensory function is only very slightly involved if C7 is involved in isolation because of the overlapping of C6 and C8. However, if one contiguous spinal nerve or trunk is involved, the muscular paralysis becomes evident. [21]

Increase in elbow flexion strength comes at the expense of increased passive moments for wrist flexion and forearm pronation caused by the increased excursions of these muscles imposed by the transfer. The only way to counteract the tendency for passive muscle forces to flex the wrist is with active wrist extension. [22]

The treatment of choice depends of the injury. In C5-C6 palsies with no elbow flexor function (grade 0–2); Steindler flexorplasty and triceps transfers have always provided good results. In C5-C6-C7 injuries, with no elbow flexor activity (grade 0), the triceps can be used if it receives a dominant innervation from C8-T1 but when the elbow flexors are grade 2, the Steindler flexorplasty is usually sufficient with active wrist extension or fused wrist.

Our study suggests that in patients with C5-C6-C7 palsy where the wrist and finger extensors are paralyzed or weaked, the flexor-pronators muscles of the forearm are strong but the triceps is not available for transfer; Steindler flexorplasty to restore elbow flexion should be complemented with wrist arthrodesis.
